# Myocardial energy metabolism in sepsis and in anemic, stagnant and hypoxic hypoxia

**DOI:** 10.1186/cc10152

**Published:** 2011-06-22

**Authors:** AJ Pereira, P Rehder, LFP Figueiredo, F Colombari, D Backer, E Silva

**Affiliations:** 1 Instituto do Coração, Universidade de São Paulo, São Paulo - SP, Brazil

## Introduction

Tissue hypoxia and inflammation are the pillars of multiple organ dysfunction. Current therapeutic interventions aimed to improve systemic oxygen delivery are mediated by increases in cardiac output, but myocardium energetic demand increases in conditions of limited supply. Only scarce data are available on heart oxygen utilization during hypoxic injuries.

## Objective

To understand the heart metabolism, challenged by different tissue hypoxia models, by examining oxygen, lactate, and glucose in vascular compartments, including coronary sinus.

## Methods

Thirty-seven pigs, fully monitored, were challenged with different injuries, including normovolemic anemia (*n *= 8), cardiac tamponade (*n *= 8), hypoxic hypoxia (*n *= 8), peritonitis-induced sepsis (*n *= 8) while five served as controls. In addition to global hemodynamics and oxygen transport, we measured oxygen saturation, lactate and glucose concentrations in arterial, pulmonary artery and coronary sinus vascular compartments. Cardiac power output was calculated as a surrogate marker of cardiac demand.

## Results

**Figure 1:**
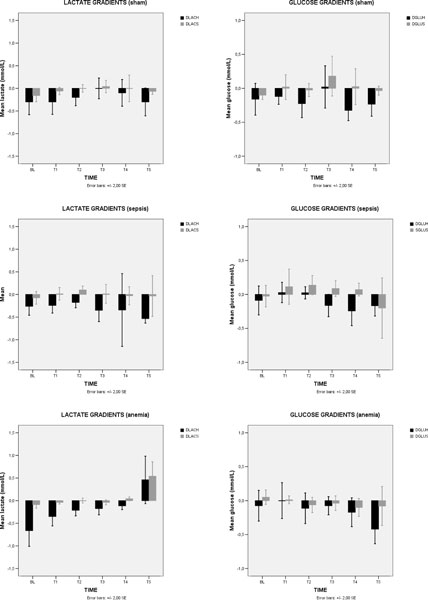
**Systemic and coronary gradients of lactate and glucose - sham, cytopathic hypoxia (sepsis) and anemic hypoxia**. DLACH = heart lactate difference = CS LAC - ART LAC; DLACS = systemic lactate difference = PA LAC - ART LAC; DGLUH = heart glucose difference = CS GLU - ART GLU; DGLUS = systemic glucose difference = PA GLU - ART GLU.

**Figure 2:**
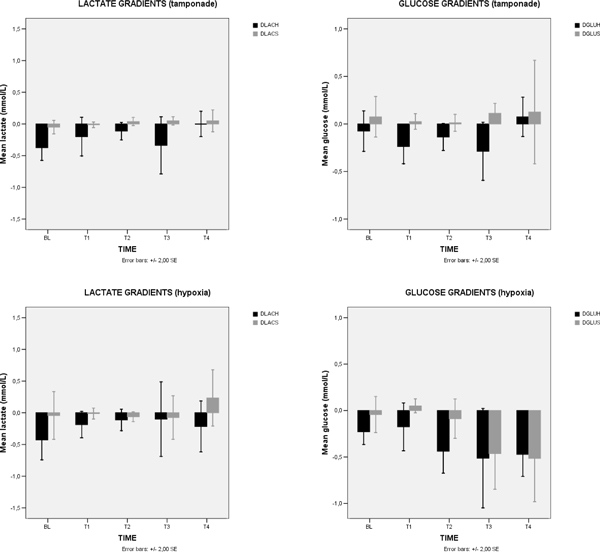
**Systemic and coronary gradients of lactate and glucose - stagnant hypoxia and hypoxic hypoxia**. DLACH = heart lactate difference = CS LAC - ART LAC; DLACS = systemic lactate difference = PA LAC - ART LAC; DGLUH = heart glucose difference = CS GLU - ART GLU; DGLUS = systemic glucose difference = PA GLU - ART GLU.

No significant alterations were found in the energetic profile in the stagnant group. There was both a decrease in lactate consumption and an increase in glucose consumption in anemia (ΔLAC changed from -0.7 to +0.5 mmol/l, *P *= 0.018; ΔGLU changed from -0.1 to -0.4 mmol/l, *P *= 0.118) and in hypoxic hypoxia (ΔLAC from -0.4 to -0.2 mmol/l, *P *= 0.361; ΔGLU from -0.25 to -0.5 mmol/l, *P *= 0.096) groups. In sepsis, we observed a progressive increase in glucose (ΔGLU from -0.1 to -0.25 mmol/l, *P *= 0.618) and lactate (ΔLAC from -0.26 to -0.53 mmol/l, *P *= 0.105) consumption by the heart. The highest lactate production was observed in late phases of anemia (+0.5 mmol/l) and the highest glucose consumption (-0.5 mmol/l) in late phases of hypoxic hypoxia. A similar and low CPO (between 3.31 and 4.4 W) was achieved in different time points according to the hypoxia model, such as a FiO_2 _about 10%, a Htc about 7%, a 30% reduction of cardiac output in tamponade, or 4 hours after fecal peritonitis induction, suggesting that the heart better tolerates hypoxia and anemia than sepsis and tamponade. See Figures 1 and 2 overleaf.

## Conclusion

Energetic substrate selection seems to be an important adaptive mechanism in response to different types of tissue oxygen delivery impairment, which may have implications on inotropic agent choice.

